# No Muscle Left Behind: Cardiac Arrest and Myocarditis in a Patient With Idiopathic Inflammatory Myopathy

**DOI:** 10.7759/cureus.72152

**Published:** 2024-10-22

**Authors:** Carson L Williams, John-Henry L Dean, Mayank Patel, Michael S Cahill, Chandra Kunavarapu, Michael Kwan

**Affiliations:** 1 Internal Medicine, San Antonio Uniformed Services Health Education Consortium, San Antonio, USA; 2 Cardiology, San Antonio Uniformed Services Health Education Consortium, San Antonio, USA; 3 Advanced Heart Failure and Cardiac Transplant Program, Methodist Heart and Lung Institute Heart Failure and Transplant Clinic, San Antonio, USA

**Keywords:** acute myopathy, focal myocarditis, fulminant myocarditis, idiopathic inflammatory myopathy, inflammatory myositis

## Abstract

Idiopathic inflammatory myopathies (IIM) are known to have extramuscular involvement, the most concerning of which is the involvement of the myocardium. Constituting a large burden of morbidity and mortality, there remains a paucity of literature describing cardiac manifestations in inflammatory myopathies, and definitive treatment and screening guidelines have yet to be published. Here, we present a rare case of cardiac arrest and fulminant myocarditis in a patient with newly diagnosed myositis.

A 71-year-old non-Hispanic White male with type 1 diabetes mellitus and hyperlipidemia presented to the rheumatology clinic with five months of progressive proximal muscle weakness and myalgias accompanied by a persistently elevated creatine kinase level and elevated liver-associated enzymes despite cessation of atorvastatin therapy three months prior. The initial examination was notable for reduced quadriceps strength bilaterally and the absence of visible skin rashes. He was found to have positive anti-Mi-2 antibody, elevated aldolase, and positive antinuclear antibody in a speckled pattern. After magnetic resonance imaging (MRI) of the left thigh demonstrated a pattern consistent with inflammatory myositis, steroid therapy was initiated, and he was referred for muscle biopsy to confirm the presumptive diagnosis of dermatomyositis. Two weeks later, before a muscle biopsy could be performed, the patient experienced a witnessed pulseless electrical activity (PEA) cardiac arrest from which he was successfully resuscitated by emergency medical services prior to hospital arrival. Subsequent cardiac evaluation showed a nonischemic cardiomyopathy with evidence of myocarditis on cardiac magnetic resonance (CMR) imaging with inferior wall hypokinesis and a left ventricular ejection fraction (LVEF) of 27%. He underwent placement of a subcutaneous implantable cardioverter defibrillator (ICD), and he responded well to intravenous immunoglobulin (IVIG), diuresis, and initiation of guideline-directed medical therapy with post-treatment transthoracic echocardiogram (TTE) demonstrating an LVEF of 40%.

This case highlights one of the rather protean and severe cardiac manifestations of IIM. Typically, the cardiac manifestations observed in IIM include subclinical electrocardiogram (ECG) and echocardiographic changes but can present, as detailed here, with fulminant myocarditis and heart failure (HF). Our purpose herein is to heighten clinician awareness of and advocate for the establishment of definitive screening and management guidelines for cardiac disease in idiopathic inflammatory myopathies.

## Introduction

Idiopathic inflammatory myopathies (IIM) are a group of rare diseases including polymyositis, dermatomyositis, and inclusion body myositis that are characterized by progressive weakness due to chronic skeletal muscle inflammation. Hardly consigned to skeletal muscle alone, this group of diseases is perhaps more appropriately classified as systemic inflammatory autoimmune diseases due to many extramuscular features, of which the poorest prognostic factor continues to be cardiac involvement despite increasing immunosuppressive therapies [1,2]. The involvement of the myocardium in the pathophysiology of these diseases has been described at length, and pathological changes observed in skeletal muscle are known to be mimicked to some degree in the myocardium [2,3]. Current knowledge predicting cardiac tissue involvement in patients with idiopathic inflammatory myopathies is threefold. These include more prevalent traditional cardiovascular risk factors, sustained involvement of the innate and adaptive immune systems with a myriad of implicated cytokines and adhesion molecules, and the well-known deleterious cardiovascular effect of chronic glucocorticoid therapy [3-7].

A systematic review involving retrospective and prospective studies broadly estimates cardiac involvement in patients with idiopathic inflammatory myopathies between 9% and 72% with wide variance attributed to the method of detection used and patient selection. Cardiac manifestations are most often subclinical and are marked by nonspecific and poorly sensitive electrocardiographic and echocardiographic changes. ST-T changes and conduction abnormalities predominate on electrocardiogram (ECG), while left ventricular hypertrophy, diastolic dysfunction, hyperdynamic left ventricular systolic function, and pulmonary hypertension predominate on echocardiographic analysis. The most prevalent symptom in IIM patients with myocardial involvement is dyspnea, a nonspecific symptom that carries a broad differential and thus requires careful attention by the diagnostician [8]. Sadly, as in the case presented here, the initial presentation can be insidious with the sudden development of fulminant myocarditis and potentially fatal arrhythmias. Interestingly, cardiac disease can manifest at the onset of myositis, develop after the initiation of treatment, and persist even after achieving myositis remission [3,9,10]. To date, no definitive screening or treatment guidelines have been made available for cardiac involvement in idiopathic inflammatory myopathies.

## Case presentation

**Figure 1:**
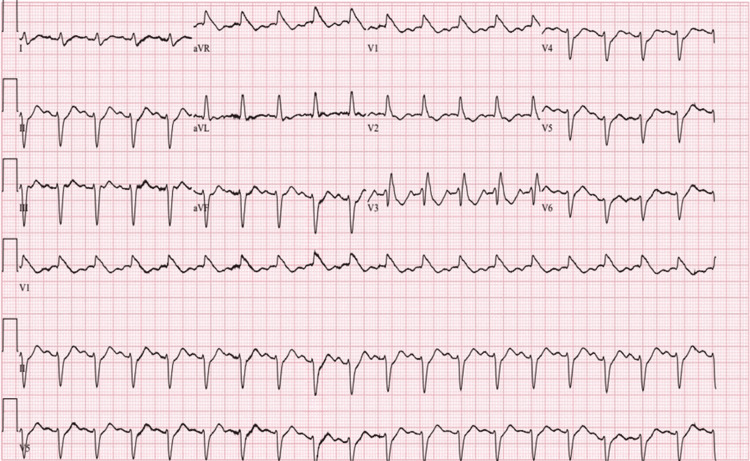
Electrocardiogram performed after arrival to the emergency room revealed sinus tachycardia with bifascicular block

A 71-year-old non-Hispanic White male with type 1 diabetes mellitus and hyperlipidemia presented to the rheumatology clinic with five months of progressive proximal muscle weakness and myalgias with consequent difficult ambulation and limited activities of daily living accompanied by a persistently elevated creatine kinase level and elevated liver-associated enzymes despite cessation of atorvastatin three months prior. The initial examination was notable for reduced quadriceps strength bilaterally and the absence of visible skin rashes. He was found to have positive anti-Mi-2 antibody, elevated aldolase, and positive antinuclear antibody in a speckled pattern. After magnetic resonance imaging (MRI) of the left thigh demonstrated a pattern consistent with inflammatory myositis, steroid therapy was initiated, and he was referred for muscle biopsy to confirm the presumptive diagnosis of dermatomyositis. Two weeks later, before a muscle biopsy could be performed, the patient experienced a witnessed sudden cardiac arrest (SCA) in the absence of preceding palpitations or chest discomfort from which he was successfully resuscitated by emergency medical services (EMS) prior to hospital arrival. Per EMS report, the initial rhythm was pulseless electrical activity followed by ventricular fibrillation with conversion to atrial fibrillation with rapid ventricular response following defibrillation. An initial electrocardiogram in the emergency room demonstrated sinus tachycardia with bifascicular block (Figure 1).

**Figure 2:**
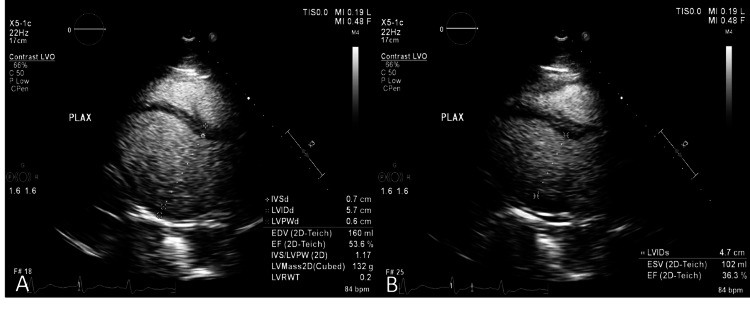
TTE parasternal long-axis view at end-diastole (A) and end-systole (B) demonstrating a reduced ejection fraction (patient had poor apical windows) TTE: transthoracic echocardiogram

**Figure 3:**
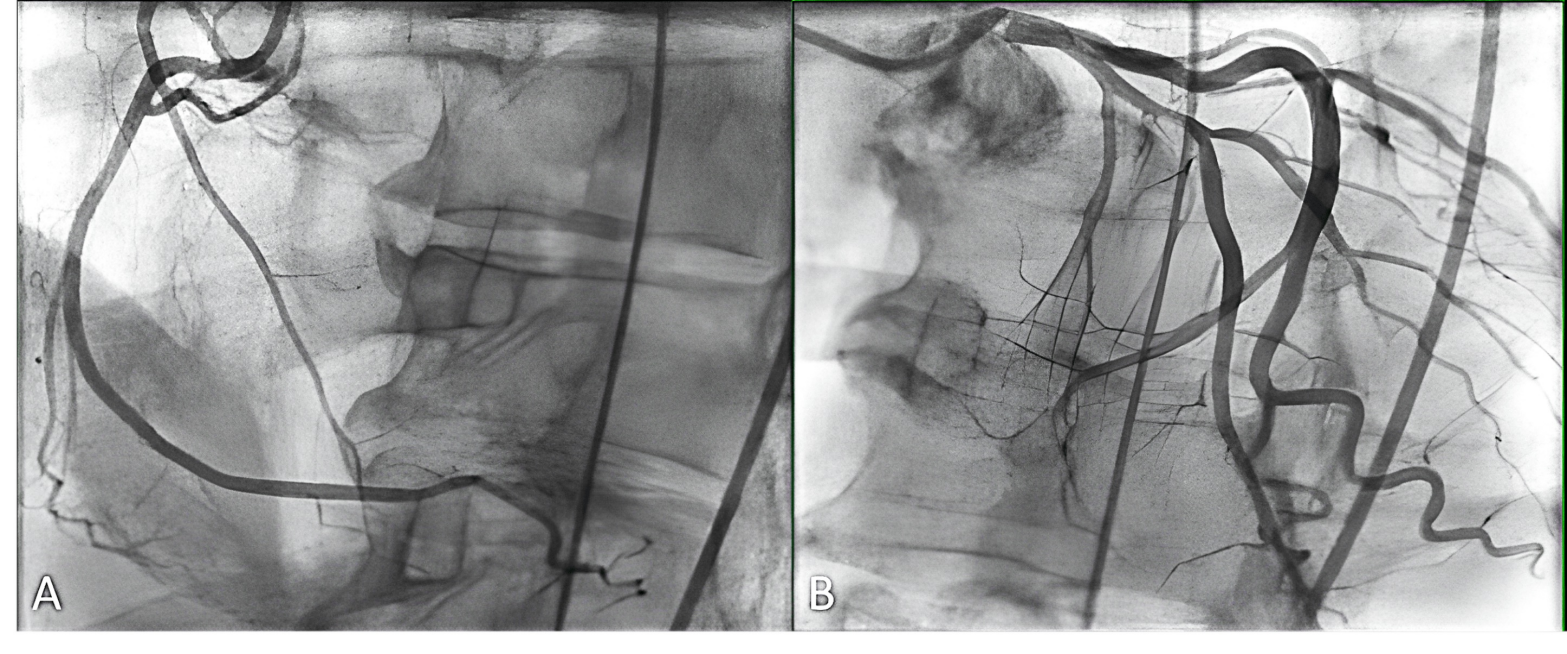
Coronary angiography displaying a codominant RCA with no significant disease (A) and moderate nonobstructive coronary atherosclerosis of the LAD and left circumflex (B) RCA: right coronary artery, LAD: left anterior descending artery

Laboratory evaluation was notable for elevated high-sensitivity troponin I (initial 1,274 ng/L and peak >125,000 ng/L before downtrend) and creatine kinase (initial 4,551 U/L with downtrend to 869 U/L). No pulmonary embolus was visualized on computed tomography pulmonary angiogram (CTPA). He was intubated and admitted to the cardiovascular intensive care unit (CVICU). On further cardiac evaluation, a transthoracic echocardiogram (TTE) demonstrated a left ventricular ejection fraction (LVEF) of 35% with diffuse hypokinesis that was most pronounced in the inferoseptal and inferior walls (Figure 2), and coronary angiography demonstrated nonobstructive coronary artery disease (CAD) (Figure 3).

**Figure 4:**
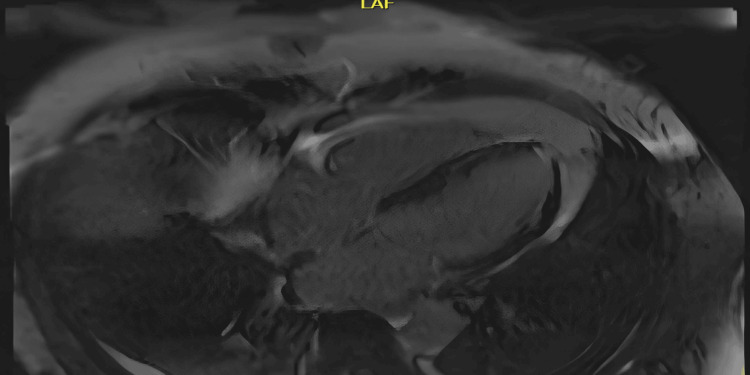
Cardiac MRI four-chamber phase-sensitive inversion recovery sequence demonstrating multifocal patchy late gadolinium enhancement due to inflammation/scarring in the mid-wall and subepicardial wall of the left ventricle involving the interventricular septum, inferolateral wall at the mid and apical level, and the true apex as well as the free wall of the right ventricle concerning for acute myocarditis MRI: magnetic resonance imaging

Cardiac MRI further revealed severely reduced biventricular systolic function (LVEF: 27%, right ventricular ejection fraction (RVEF): 25%) and multifocal patchy late gadolinium enhancement suggestive of inflammation and fibrosis, findings consistent with acute myocarditis (Figure 4).

Subsequent electrocardiograms would demonstrate resolution of ST elevation observed in V1 on the initial electrocardiogram. Secondary prevention of sudden cardiac arrest (SCA) was pursued with the placement of a subcutaneous implantable cardioverter defibrillator (ICD). Further diagnostic clarification with an endomyocardial biopsy was deferred, and empiric therapy was initiated with brisk clinical response to intravenous immunoglobulin (IVIG), diuresis, and initiation of guideline-directed medical therapy for nonischemic cardiomyopathy with post-treatment TTE showing an ejection fraction of 40%.

## Discussion

Cardiac manifestations of idiopathic inflammatory myopathies (IIM) are diverse and unpredictable. Most frequently, cardiac manifestations in IIM involve subclinical electrocardiogram (ECG) and echocardiographic changes. Unfortunately, in our patient's case, the first sign of cardiac involvement was cardiac arrest secondary to fulminant myocarditis.

Myocardial involvement in IIM is well documented in the literature, primarily in the form of case reports, case series, and cohort studies, but clinical practice-changing conclusions have yet to be drawn. Myocardial involvement constitutes a significant degree of morbidity and mortality in IIM patients; indeed, one study found cardiovascular manifestations to be the most common cause of death [1]. Gupta et al., in a systematic review, attempted to quantify cardiac involvement in IIM [11]. Dysrhythmias were seen in 31.8% of electrocardiograms, and 34.5% of patients were found to have diastolic dysfunction on echocardiographic assessment. Congestive heart failure (HF) was the most common cause of death, accounting for 21% of total cardiac mortality. On review of pathology literature, 38% of patients were found to have myocarditis, and 42% were found to have myocardial fibrosis [11]. These findings may implicate the need for inpatient and extended cardiac monitoring as well as early initiation of guideline-directed medical therapy to improve outcomes in this patient population.

Increasingly sensitive diagnostic techniques have heightened awareness to a greater prevalence of cardiac involvement in IIM than previously thought. Cardiac magnetic resonance (CMR) imaging has emerged as the foremost technique. In a recent case series of 53 patients with histologically proven IIM and absence of cardiac symptoms, 62.3% of patients were found to have signs of myocardial inflammation via CMR assessment [12].

Known to be a poor prognostic indicator with nearly 70% of affected patients only having subclinical involvement [13,14], this case underscores the need for improved definitive screening and management recommendations for cardiac disease in IIM. To that end, a recent case-control study by Huang et al. sought to explore the utility of a multiparametric cardiac magnetic resonance (CMR) score in screening for early cardiac involvement in IIM compared with the clinical Myositis Disease Activity Assessment Tool (MDAAT) [15]. Late gadolinium enhancement on CMR, while highly specific in detecting focal myocardial fibrosis, may miss early cardiac involvement in IIM in the form of diffuse fibrosis. Utilizing native T1 and T2 mapping, the authors found evidence of diffuse myocardial fibrosis in patients with IIM compared to healthy controls. With further validation and a larger sample size, this newly developed CMR score could identify subclinical cardiac involvement with higher sensitivity and facilitate earlier intervention, potentially reducing morbidity and mortality in IIM [15].

Treatment recommendations in acute myocarditis are non-standardized and involve attempts at achieving inflammation quiescence through the use of empiric steroids with or without additional immunosuppressive agents. Historical data has been conflicting at best. The Myocarditis Treatment Trial demonstrated no clear benefit to immunosuppression, although the initiation of therapy was delayed in the studied population for as long as one year from symptom onset [16]. A later case series implementing therapeutic methylprednisolone followed by cyclophosphamide or azathioprine demonstrated efficacy with improved cardiac clinical symptoms and reduced myocardial inflammation as evidenced by CMR [17]. Most recently, in the Tailored Immunosuppression in Inflammatory Cardiomyopathy (TIMIC) trial, in which 85 patients with virus-negative chronic inflammatory cardiomyopathy were randomized to a six-month course of prednisone plus azathioprine or standard heart failure (HF) medications, the immunosuppressive arm was found to have a significant and durable reduction in symptoms and improvement in LV function comparatively [18]. Larger prospective and randomized trials would aid in establishing definitive guidelines.

## Conclusions

Cardiac involvement, primarily in the form of arrhythmias, heart failure, and myocarditis, in IIM is well known and constitutes a large degree of morbidity and mortality, but the relative rarity of the disease itself and a paucity of data has precluded the establishment of definitive screening and therapeutic guidelines. CMR has emerged as an innovative imaging modality and has demonstrated subclinical detection of myocardial fibrosis in patients with IIM, suggesting a potential screening role for CMR in these patients. Multimodal screening techniques in this patient population may permit subclinical detection of disease, facilitate earlier intervention, and improve outcomes. Treatment recommendations in myocarditis are often centered around immunosuppression with corticosteroids with or without additional immunomodulating agents. Although historical data confirming immunosuppressive efficacy was lacking, newer randomized data demonstrates durable immunosuppressive efficacy in chronic virus-negative inflammatory cardiomyopathy. Future directions of research should be aimed at further clarification of the extent of cardiac involvement in IIM, determining the necessity and optimal modality of screening patients with IIM for myocardial involvement, and standardization of the therapeutic approach to the management of acute myocarditis.
